# Comparison of artificial intelligence-generated and physician-generated patient education materials on early diabetic kidney disease

**DOI:** 10.3389/fendo.2025.1559265

**Published:** 2025-04-22

**Authors:** Miaomiao Cheng, Qi Zhang, Hua Liang, Yanan Wang, Jun Qin, Lei Gong, Sha Wang, Luyao Li, Xiaoyan Xiao

**Affiliations:** ^1^ Qilu Hospital of Shandong University, Department of Nephrology, Jinan, Shandong, China; ^2^ Healthcare Big Data Research Institute, Cheeloo College of Medicine, Shandong University, Jinan, Shandong, China; ^3^ Qilu Hospital of Shandong University, Department of Endocrinology, Jinan, Shandong, China

**Keywords:** diabetes, diabetic kidney disease, artificial intelligence, large language models, patient education

## Abstract

**Background:**

Diabetic kidney disease (DKD) is a common and serious complication of diabetes mellitus and has become the most important cause of end-stage renal disease (ESRD). In light of the rising prevalence of diabetes, there is a growing imperative for the early detection and intervention of DKD. With the rapid development of artificial intelligence (AI) technologies, its potential applications in patient education are receiving increasing attention, especially large language models (LLMs). The aim of this study was to evaluate the quality of LLMs-generated patient education materials (PEMs) for early DKD and to explore its feasibility in patient education.

**Methods:**

Four LLMs (ERNIE Bot 4.0, GPT-4o, ChatGLM4, and ChatGPT-o1) were selected for this study to generate PEMs. Among them, ERNIE Bot 4.0, GPT-4o, and ChatGLM4 generated 2 versions of PEMs based on American Diabetes Association(ADA) guidelines and without ADA guidelines, respectively. ChatGPT-o1 only generated a PEM without ADA guidelines. An experienced physician wrote a PEM based on ADA guidelines. All materials were assessed using a Likert scale which covered the dimensions of accuracy, completeness, safety, and patient comprehensibility. A total of 7 medical experts (including nephrologists and endocrinologists) and 50 diabetic patients were invited to evaluate the study. We recorded basic information on the patient evaluators.

**Results:**

Experts evaluated PEMs from ERNIE Bot 4.0, GPT-4o, ChatGLM4, and ChatGPT-o1, plus physician-sourced PEM. Results showed ERNIE Bot 4.0’s non-guideline PEM and physician-sourced PEM were the top two. Patient assessments of the 2 top-scoring PEMs found that the ERNIE Bot 4.0’s non-guideline PEM performed as well as, if not slightly better than, the physician-sourced PEM in terms of patient comprehensibility, completeness, and safety. In addition, the non-guideline-based PEM was preferred for patients with a history of diabetes longer than 5 years and for patients with proteinuria. Surprisingly, GPT-4o and ChatGLM4’s non-guideline PEMs outperformed guideline-based ones.

**Conclusion:**

The LLMs-sourced PEMs, especially the ERNIE Bot 4.0’s non-guideline PEM for early DKD, performed comparably to the physician-sourced PEM in terms of accuracy, completeness, safety, and patient comprehensibility, and exerted a high degree of feasibility. AI may show the potential for broader applications in patient education in the near future.

## 1 Introduction

Diabetic kidney disease (DKD) is one of the most common complications of diabetes and a leading cause of end-stage renal disease (ESRD). In 2022, an estimated 828 million adults worldwide had diabetes, a significant increase of 630 million from 1990, increasing the global burden of diabetes (1). This significant rise in the global prevalence of diabetes has greatly exacerbated the burden of related complications, including DKD. With the increasing number of diabetic patients, the prevalence of DKD has also risen, posing an escalating public health challenge.

Globally, approximately 30%-50% of ESRD patients have diabetes (2). Early-stage DKD often has no obvious clinical symptoms, which may lead to missed opportunities for timely intervention. Early recognition and intervention are therefore critical to slowing the progression of the disease, which is closely associated with chronic hyperglycemia, inadequate blood pressure control, and unhealthy lifestyle. Effective early prevention can greatly slow the progression of the disease, reduce the risk of developing ESRD, and ultimately reduce the need for dialysis or kidney transplantation, thus improving the quality of life of patients.

In clinical practice, we have found that many diabetic patients have inadequate knowledge about DKD. Therefore, in addition to clinical screening and intervention by healthcare providers, patient education plays a crucial role in DKD management. However, effective patient education requires a more efficient and personalized approach to improve their level of cognition. Patient education materials (PEMs) are core tools for chronic disease management that increase knowledge, patient engagement, and treatment adherence, and can lead to better overall outcomes (3). However, PEMs are costly and require a lot of time to develop and update (4). In this field, the application of artificial intelligence (AI) offers a new solution for early DKD patient education. In recent years, the application of AI technology in the medical field has become increasingly widespread, with growing interest in the potential of large language models (LLMs), such as ChatGPT, to support patient education. Examples include the performance of ChatGPT-4 and Google Bard in generating educational materials for patients undergoing cataract surgery (5) and the study of ChatGPT-4 and Google Bard in generating educational materials for patients with obstructive sleep apnea (6) and so on. LLMs have shown great promise in creating PEMs (7). However, the accuracy, safety, completeness, and comprehensibility of PEMs generated by LLMs for early-stage DKD have yet to be thoroughly evaluated.

This study aims to assess the quality of early-stage DKD PEMs generated by LLMs and explore their feasibility as a tool for educating diabetic patients at risk of kidney disease.

## 2 Materials and methods

### 2.1 Ethics and design

The study was approved by the Ethics Committee of Qilu Hospital, Shandong University, with the ethical number of KYLL-202502-043-1. All participants gave informed consent and data were collected and processed in an ethical manner.

### 2.2 Material sources and processing

A physician-generated PEM was created by a nephrologist with over 10 years of experience in DKD research. The PEM was based on the 2024 Standards of Care in Diabetes from the American Diabetes Association (ADA) guidelines, specifically the section on chronic kidney disease (CKD). Chinese patients were included in this study, and to ensure the generalizability of the findings across both Chinese and English contexts, we selected a range of advanced language models. These included ERNIE Bot 4.0, ChatGLM4, GPT-4o, and ChatGPT-o1. To ensure consistency, all LLMs generated their PEMs on September 29, 2024. 4 LLMs were selected for this task. We used the web version for all 4 models. Each model was instructed to generate content resembling a doctor’s response, without disclosing its AI identity. The models were also specifically asked to respond as nephrology experts. Additionally, the ADA guidelines in PDF format were uploaded for ERNIE Bot 4.0, GPT-4o, and ChatGLM4. Before inputting the prompt to the LLMs, we required that the 3 LLMs, ERNIE Bot 4.0, GPT-4o, and ChatGLM4, have successfully read the ADA Guidelines attachment that we uploaded (the attachment is named “ame Guidelineslyl and then we required them to generate PEMs based on the ADA Guidelines according to the prompt. Since the ChatGPT-o1 does not support uploading documents, we generate the science material via prompt only. The PEM created by the physician followed the same set of instructions and questions. Detailed prompts are provided in **Supplementary Table 1** of the **Supplementary Materials**. All materials were crafted to meet the reading level of a 6th-grade education, as recommended by the American Medical Association (AMA) (8). Standardized formatting was applied to all generated materials, using a consistent font and size.

### 2.3 Assessments of PEMs

**Figure 1:**
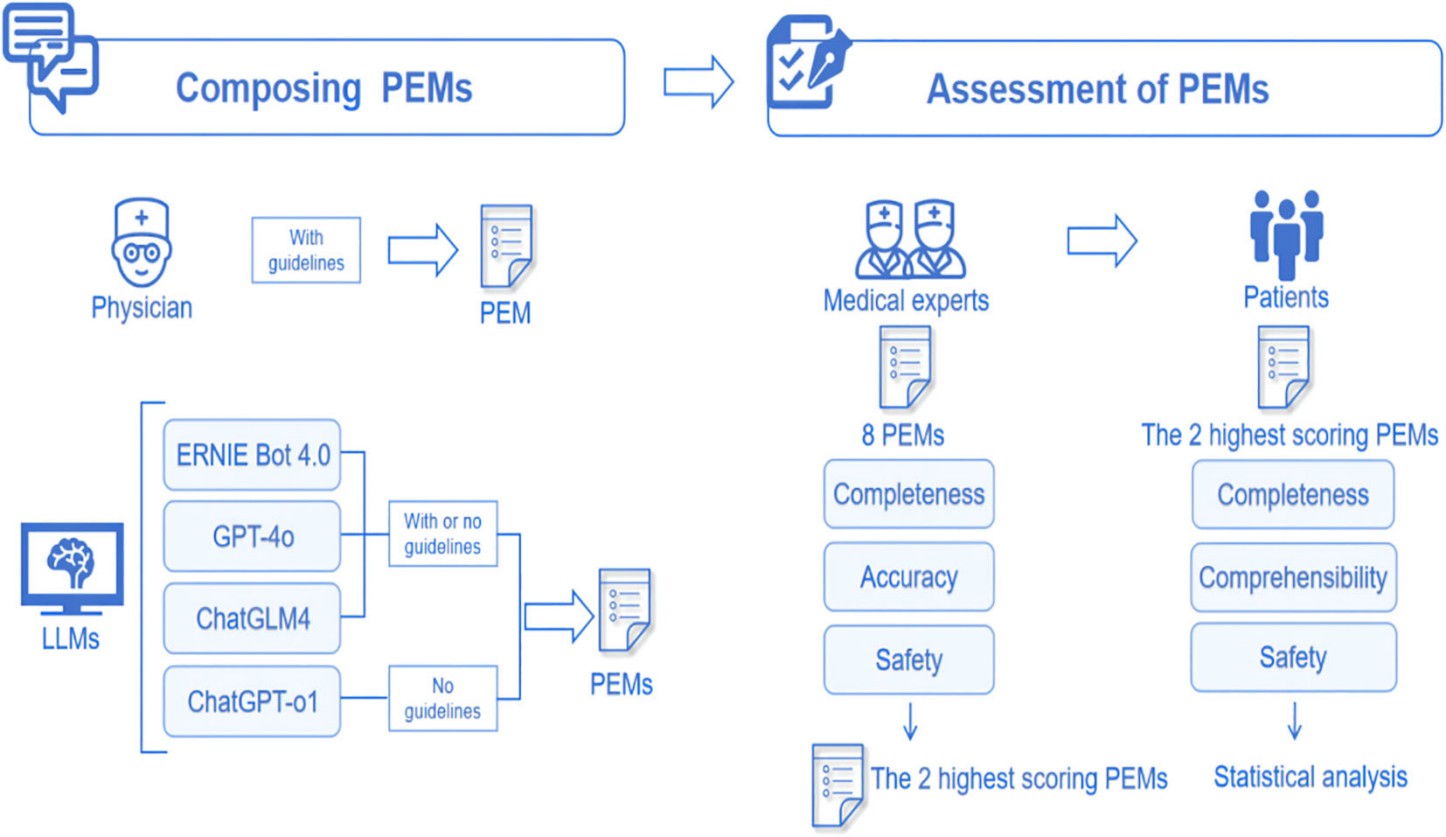
The workflow of this study.

All PEMs underwent professional evaluation. We assembled a review panel consisting of 7 experts in nephrology and endocrinology, each with a minimum of 5–10 years of clinical experience in the management of DKD. The experts conducted their evaluations in a blinded manner, meaning they did not know whether the PEM was physician-sourced or generated by LLMs. The evaluation was based on the Likert scale, covering 3 dimensions: accuracy, completeness, and safety. Based on the pre-experimental data, a medium effect size was assumed (Cohen’s d = 0.5, PS = 0.65), the significance level was set at α = 0.05, and statistical efficacy was set at 1-β = 0.8, and after performing the efficacy analyses, 50 patients were ultimately enrolled in the evaluation. We then selected the 2 PEMs with the highest scores from expert assessments and invited 50 people with diabetes to assess and score their comprehensibility, completeness, and safety. We collected demographic data including age, gender, occupation, type of diabetes, duration of diabetes, and presence of proteinuria in all patients. The evaluation of accuracy used a six-point Likert scale, while completeness and safety were assessed using a three-point Likert scale. Patient comprehensibility was rated using a five-point Likert scale. The specific assessment questions employed in this study are provided in **Supplementary Table 2** of the **Supplementary Materials**. The whole flow chart is shown in **Figure 1**.

### 2.4 Statistical analysis

Categorical variables in this study were described using frequencies and percentages. Continuous variables were first assessed for normality using the Shapiro-Wilk test. Results of expert assessments results were assessed using Friedman’s test followed by Dunn’s multiple comparisons test for describing differences between PEMs. Results of patient assessments and when comparing differences between guideline-based and non-guideline-based generated PEMs were assessed using the paired t-test for variables that fit a normal distribution; the Wilcoxon signed-rank test was used for variables that did not fit a normal distribution. Data were analyzed using GraphPad Prism 9.5.1 (GraphPad Software, San Diego, CA, USA) and SPSSAU (Version 25.0). A *p*-value of < 0.05 was considered statistically significant.

## 3 Results

### 3.1 Basic characters of evaluators

All nephrologists and endocrinologists involved in the evaluation had an M.D. or Ph.D. degree and extensive clinical work experience. A total of 50 patients participated in the evaluation, with 56% (28/50) being male. The average age of the patients was 43.26 years. In terms of age distribution, 14% (7/50) of the patients were between 14 and 30 years old, 52% (26/50) were between 31 and 50 years old, and 34% (17/50) were over 50 years old. Regarding occupational background, 24% (12/50) of the patients were farmers, 16% (8/50) were office workers, 12% (6/50) were government employees, 10% (5/50) were professionals or technical personnel, 6% (3/50) were business managers, and 6% (3/50) were students. The remaining 26% of patients were engaged in other occupations, including manual laborers, freelancers, and retirees. With respect to diabetes diagnosis, 82% (41/50) of the patients had a confirmed diagnosis of type 2 diabetes, 16% (8/50) had type 1 diabetes, and 1 patient was diagnosed with latent autoimmune diabetes in adults (LADA). In terms of duration of diabetes, 25 (50%) patients had a duration of 5 years or more, and 25 (50%) had a duration of less than or equal to 5 years. Additionally, 18% (9/50) of the patients had proteinuria.

### 3.2 Expert evaluations of all PEMs

**Figure 2:**
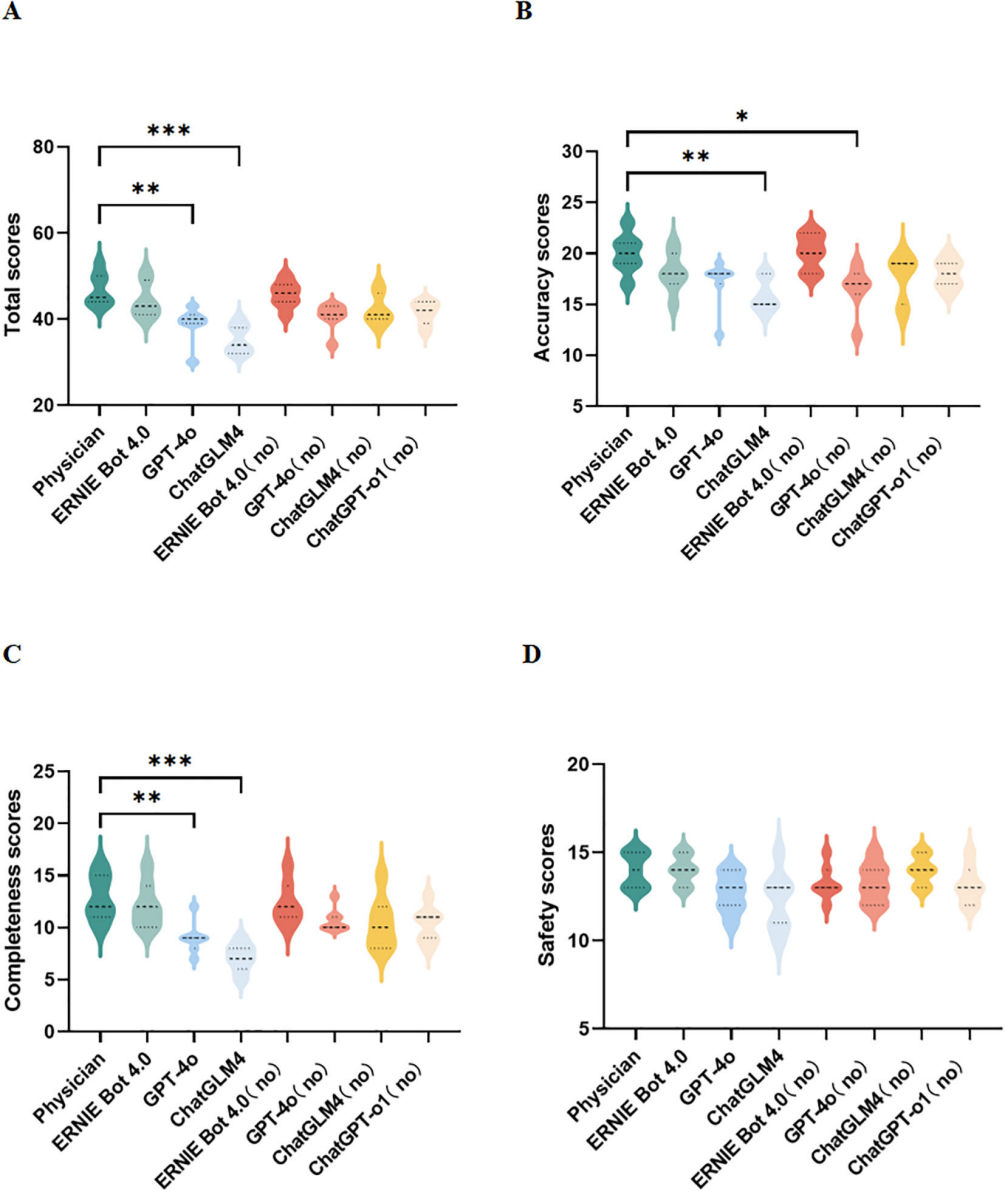
Expert assessments. **(A–D)** Comparison of total, accuracy, completeness, and safety scores for different PEMs; no, no guidelines; *p < 0.05, **p < 0.01, ***p < 0.001, ****p< 0.0001.

In terms of the total scores, the PEMs generated by ERNIE Bot 4.0 are comparable to the physician-sourced PEM, the PEMs generated by other LLMs generally had lower scores than the physician-sourced PEM(**Figure 2A**). In terms of accuracy, the accuracy of PEMs generated by LLMs was acceptable with the exception of PEM generated by ChatGLM4 (guideline-based version) (**Figure 2B**). Of all the PEMs, the PEM generated by ERNIE Bot 4.0 (non-guideline-based) had the highest accuracy, followed by the physician-sourced PEM (**Figure 2B**). In terms of completeness, PEMs generated by the 2 LLMs, GPT-4o (*p*=0.0050) and ChatGLM4 (*p* = 0.0001), based on the guidelines were significantly different from the physician-sourced PEM (**Figure 2C**). In terms of safety, there was no significant difference in safety between all PEMs, including those generated by LLMs and that sourced by the physician (**Figure 2D**). All experts agreed that these PEMs do not cause any harm to patients. In our study, 2 PEMs from ERNIE Bot 4.0 (non-guideline-based) and the physician performed better in terms of accuracy, completeness, and safety.

### 3.3 Patient evaluations of PEMs generated by ERNIE Bot 4.0 and physician

**Figure 3:**
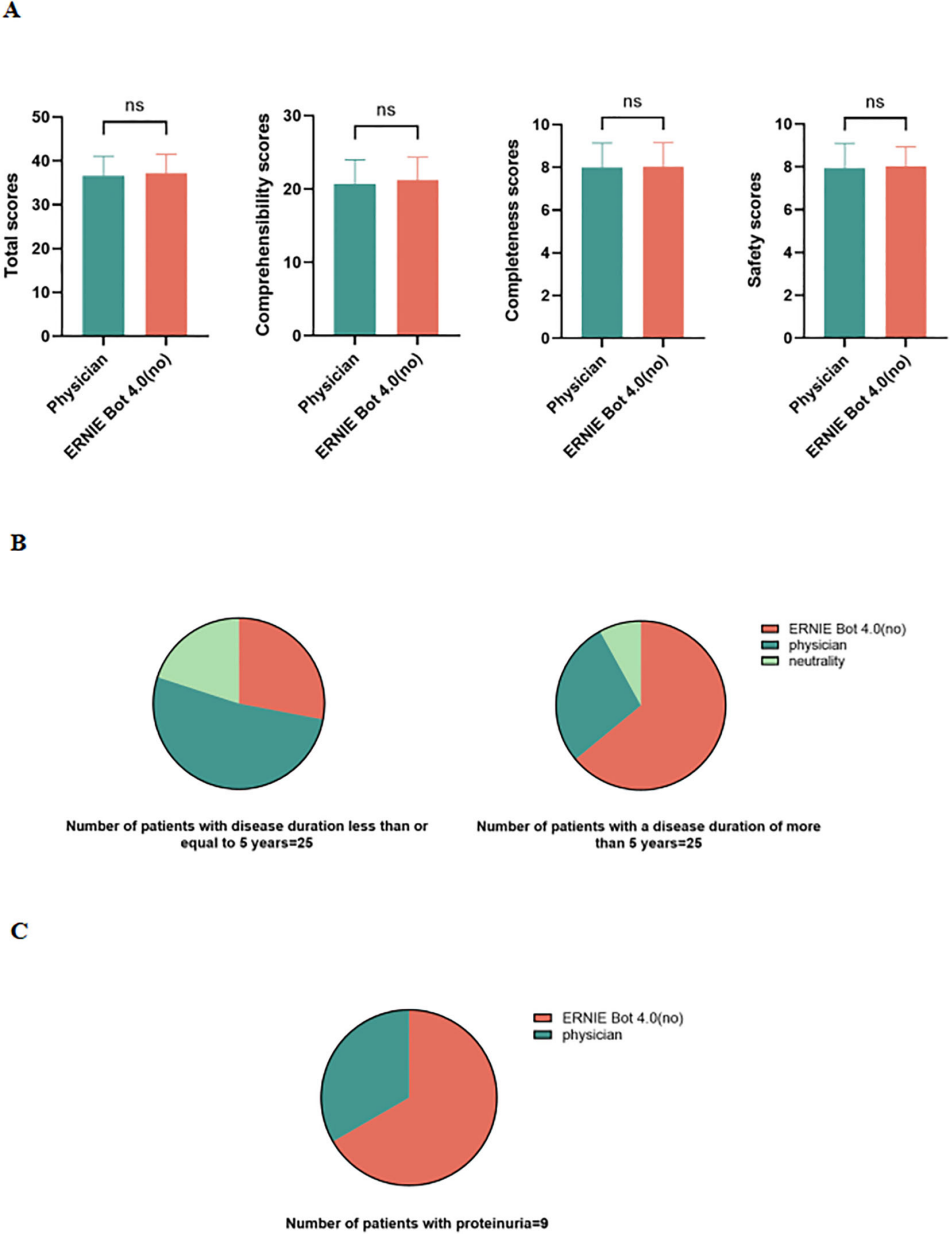
Patient assessments. **(A)** Comparison of 2 PEMs in terms of total scores, patient comprehensibility, completeness, and safety scores; **(B)** Assessments of 2 PEMs in patients with different disease duration; **(C)** Assessments of 2 PEMs in patients with proteinuria; ns, not significant.

Because 2 PEMs from the physician and ERNIE Bot 4.0 (non-guideline based) scored highest in expert assessments, we performed patient assessments of ERNIE Bot 4.0 non-guideline-based generated PEM against the physician-sourced PEM. Patient assessments were similarly conducted in a blinded manner. We found that ERNIE Bot 4.0 performed on par with physicians in terms of patient comprehensibility, completeness, and safety. 60% (30/50) of patients felt that ERNIE Bot 4.0 performed as well or better than the physician. In fact, the non-guideline-based version of the PEM generated by ERNIE Bot 4.0 even scored slightly higher than the physician-sourced PEM (**Figure 3A**).

Additionally, in a survey of a group of patients with a disease duration of more than 5 years, 64% (16/25) thought that the non-guideline-based version of the PEM (patient education material) generated by ERNIE Bot 4.0 was superior to the physician-provided PEM, whereas 28% (7/25) preferred to think that the physician-provided PEM was more effective, and another 8% (2/25) were neutral (**Figure 3B**). In contrast, among patients with a disease duration of less than or equal to 5 years, 52% (13/25) thought that the physician-provided PEM was superior, while 28% (7/25) tended to think that the non-guideline-based version of the PEM generated by ERNIE Bot 4.0 was better, and an additional 20% (5/25) were neutral on both (**Figure 3B**).

In addition, 9 of the 50 participating patients had proteinuria, and 6 of these patients gave higher scores to the PEM generated by ERNIE Bot 4.0 (**Figure 3C**).

### 3.4 Comparison of guideline-based and non-guideline-based PEMs

**Figure 4:**
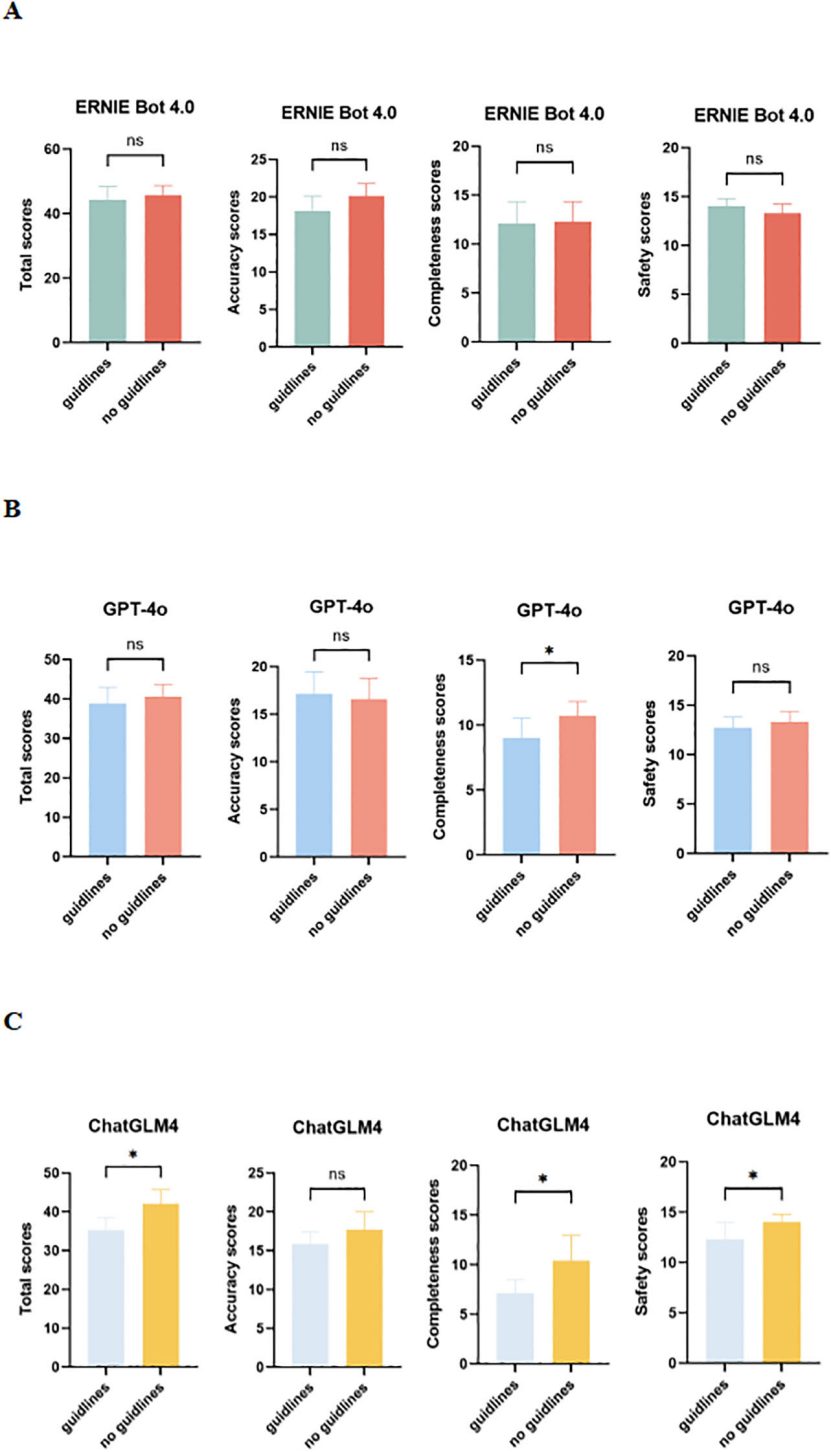
Comparison of guideline-based and non-guideline-based versions of PEMs generated by LLMs in expert assessments. **(A)** Total, accuracy, completeness, and safety scores for 2 versions of PEMs generated by ERNIE Bot 4.0; **(B)** Total, accuracy, completeness, and safety scores for 2 versions of PEMs generated by GPT-4o; **(C)** Total, accuracy, completeness, and safety scores for 2 versions of PEMs generated by ChatGLM4; ns, not significant. *p < 0.05, **p < 0.01, ***p < 0.001, ****p < 0.0001.

The evaluation results of the two versions of PEMs based on the guidelines and those not based on the guidelines generated by LLMs (ERNIE Bot 4.0, GPT-4o, and ChatGLM4) are presented in **Figure 4**. There is no difference in accuracy, completeness, or safety between the two versions of PEMs generated by ERNIE Bot 4.0 (**Figure 4A**). For the 2 versions of PEMs generated by GPT-4o, differences were observed in terms of completeness (*p* = 0.0156) (**Figure 4B**). Interestingly, in our study, LLMs performed worse with guidelines than without guidelines. Compared to the guideline-based PEM, ChatGLM4 performed somewhat better when the PEM was not generated based on the guidelines, especially in terms of completeness (*p* = 0.0213) and safety (*p* = 0.0300), which may be related to the ability of the models to process long texts (9) (**Figure 4C**).

## 4 Discussion

Early diagnosis and intervention are essential to slow down the progression of DKD; therefore, patient education plays a key role in the management of early DKD (10). As artificial intelligence evolves, its potential for patient education comes under scrutiny. To assess the accuracy, completeness, safety, and comprehensibility of LLMs in generating PEMs for early DKD, 4 LLMs (ERNIE Bot 4.0, GPT-4o, ChatGLM4, and ChatGPT-o1) were selected to generate PEMs under the same conditions, including guideline-based and non-guideline-based versions. These PEMs were then blindly evaluated by a group of medical experts, along with the physician-sourced PEM. The results showed that experts considered the PEMs generated by all LLMs to be relatively well-accepted in terms of accuracy and safety. In addition, 50 patient evaluators were unknowingly assessed on the 2 PEMs that scored highest in expert assessments. The 2 PEMs were from the physician and ERNIE Bot 4.0 (non-guideline-based). The results show that the PEM generated by ERNIE Bot 4.0 (non-guideline-based) was comparable to, or even slightly better than, the physician-sourced PEM on some rating dimensions in patient assessments. Our findings suggest that LLMs have great potential for generating high-quality PEMs. This is in line with existing literature, where AI techniques can play an important role in patient education (7). Our study utilized a dual approach that included both expert and patient assessments, which is different from previous studies that focused only on expert or patient assessments.

Interestingly, in patient assessments, we found that acceptance and preference for PEMs may vary between patient groups. For example, patients with longer disease duration or those experiencing proteinuria showed a stronger preference for the PEM generated by the LLM. ERNIE Bot 4.0, in particular, achieved a high level of acceptance among patients with longer disease duration. This could be related to the AI’s ability to provide personalized, accurate, and timely content, along with the efficiency of generating diverse educational materials quickly. In contrast, patients with shorter disease duration were more likely to prefer the physician-sourced PEM. However, due to the relatively small sample size, further studies with larger patient cohorts are necessary to assess the broader applicability and value of LLM-generated PEMs in clinical settings. Based on these results, when designing PEMs in the future, we can adjust the educational materials according to the patient’s background, such as adjusting the level of linguistic complexity, visual design, and difficulty of the content, so as to improve the accessibility and usefulness of the PEMs. Patient feedback can also be incorporated into the content creation process, allowing LLMs to evolve and deliver more effective PEMs. This dynamic patient-centered approach ensures that information is presented in a way that is most appropriate for each individual.

In addition, we observed differences in integrity and safety between guideline-based PEMs and non-guideline-based PEMs, especially those generated by GPT-4o and ChatGLM4. One possible explanation for the underperformance of the guideline-based LLMs could be the complexity of the PDF format, challenges related to text extraction, and the potential loss of context (11). Parsing complex medical documents often requires advanced contextual understanding and information extraction capabilities, which are areas where LLMs still face significant challenges (12). The possibility for LLMs to misinterpret or omit crucial information can lead to incomplete or inaccurate content. To address these issues, future research should focus on improving LLMs’ performance in understanding long-form documents and extracting critical information. Incorporating advanced techniques such as named entity recognition and relationship extraction could enhance the model’s ability to process complex, multi-paragraph medical texts (13).

AI technology has demonstrated considerable potential in medical education, not only improving the quality of education but also facilitating personalized learning. In addition, AI can enhance educational effectiveness by streamlining assessment and feedback processes in medical education (14). In clinical decision support and intelligent question-and-answer systems, AI applications have significantly improved healthcare efficiency, contributing to overall improvements in healthcare service quality (15). Studies by Wang et al. and others have shown that LLM-generated information, such as patient profiles for kidney stone treatment, can be highly accurate with minimal omissions (16). In addition, research in primary diabetes care and diabetic retinopathy (DR) screening has demonstrated significant improvements in patient self-monitoring and adherence through the use of LLMs (17). Our study adds to this body of evidence and further confirms the potential of LLMs in patient education, with both expert and patient evaluations showing positive results.

Our study highlights the potential of AI in educating patients with DKD and suggests that in some cases AI may be superior to traditional physician-led education. Traditional physician-generated educational materials often adopt a “one-size-fits-all” approach, which may not be suitable for diverse patient populations with varying needs and comprehension (18). For instance, cultural factors can influence patient comprehension and engagement with health information (19). LLMs can overcome this limitation by offering more personalized content, not only addressing disease management but also providing advice on diet, exercise, and emotional support (20). By generating customized PEMs, LLMs can improve patient understanding, increase motivation for treatment adherence, and enhance the overall patient education experience.

Management of DKD requires not only pharmacological interventions but also ongoing patient self-management in terms of diet, exercise, and glucose monitoring (21). To avoid missed treatment opportunities due to patient misinterpretation of PEMs during self-management, we need to ensure that PEMs generated by LLMs for patients with DKD are accurate, clear, and clinically credible. However, LLMs are still susceptible to the problem of ‘hallucination’, where they may generate responses that appear reasonable but are factually incorrect or incomplete (22, 23). This issue is of particular concern in healthcare contexts where the accuracy of information is paramount. Additionally, LLMs sometimes use assertive language that may lead patients to place undue trust in potentially inaccurate information (24). To mitigate the risks of misinformation, we recommend the implementation of safeguards, such as content proofreading and expert review, to detect and correct errors in a timely manner. AI tools can be developed to automate the cross-validation of AI-generated medical information against the latest clinical guidelines, academic research, specialized medical databases, and reputable medical journals. For instance, AI-generated content could be cross-referenced with authoritative sources, such as the American Diabetes Association (ADA) guidelines for diabetes management, the Improving Global Prognosis in Kidney Disease (KDIGO) guidelines for chronic kidney disease (CKD) management, and the International Society of Nephrology (ISN) guidelines for diabetic kidney disease (DKD). This process would help identify potential inconsistencies or discrepancies in educational materials, ensuring their accuracy and alignment with current clinical standards. In addition, to improve the accuracy, clarity, and completeness of the PEMs further, we propose to train these models on the DKD-specific dataset. Such specialized datasets can be created by collecting high-quality data from medical literature, clinical cases, and patient feedback, enabling the models to generate more accurate and contextually relevant PEMs for DKD patients. A similar approach in ophthalmology has shown that training LLMs on specialized datasets can improve accuracy in generating content related to that specialty (25). The diversity and quality of training datasets are directly related to the validity and reliability of LLM-generated PEMs. It is therefore essential to ensure that training data includes cases from different stages of disease progression and relevant clinical guidelines. We propose the establishment of a public registry or database to track and report potential errors or gaps in DKD-related educational content. Such a platform would provide valuable feedback for improving LLM performance, ensuring that misinformation is minimized, and enabling the identification and correction of medical factual errors, outdated guidelines, and misinterpreted treatment recommendations. This transparent, trackable system will be essential in advancing the development of reliable LLMs for clinical education and improving the quality of patient care. Nevertheless, LLM-generated PEMs may still convey incomplete medical information, which may lead to a biased understanding of the disease process in patients with DKD. Differences in the training datasets of different models may lead them to provide different professional advice (26), and their completeness may in some cases not meet the medical standards required for patient education. Therefore, we recommend that LLMs automatically generate disclaimers that make it clear that the information provided is for informational purposes only and should not be used as a substitute for professional medical advice. We also recommend protecting against potential legal risks associated with misinformation.

Several limitations of this study should be acknowledged. First, all cases were sourced from a single medical center. This may limit the generalizability of the findings. Additionally, the small sample size constrained the ability to detect significant differences between groups. Future studies should incorporate multicenter designs and larger, randomized samples to better validate these findings. Moreover, the scoring system used in this study was somewhat subjective and lacked comprehensive assessment criteria. To address this, we recommend the development of a standardized system for evaluating educational materials for DKD patients. In addition, a single PEM from a physician source may limit the generalizability of the findings, but it follows existing guidelines and is consistent with materials widely used in clinical practice at our institution. If feasible, in the future we would be willing to include additional physician-generated PEMs for comparison or perform sensitivity analyses to enhance the reliability of the results. Finally, the absence of a widely recognized Chinese readability assessment tool limited the ability to provide a comprehensive and objective evaluation of the readability of the materials in the study. Future research should aim to create or adopt an established tool for readability assessment in Chinese to ensure a more robust evaluation of educational materials.

## Data Availability

The original contributions presented in the study are included in the article/**Supplementary Material**. Further inquiries can be directed to the corresponding author.
