# Supplementary material for: Comparison of artificial intelligence-generated and physician-generated patient education materials on early diabetic kidney disease
Source: Front Endocrinol (Lausanne). 2025 Apr 22;16:1559265. doi: 10.3389/fendo.2025.1559265 (PMC12052532; doi:10.3389/fendo.2025.1559265)
Supplement: Supplementary file 1 [file DataSheet1.docx]

Supplementary Material

# Supplementary Tables

| **Supplementary Table 1. Detailed prompts for large language models.**   \| LLMs \| ERNIE Bot 4.0 \| Not based on guidelines \| You are now a nephrologist, and you need to write an educational handout for patients. The handout should answer the following questions and be written at a sixth-grade reading level. 1.What is early diabetic kidney disease? 2.What are the early signs of diabetic kidney disease? 3.How is diabetic kidney disease treated? 4.What are the risk factors for diabetic kidney disease? 5.How can diabetic kidney disease be prevented? \| \| --- \| --- \| --- \| --- \| \| GPT-4o \| \| ChatGLM4 \| \| ChatGPT-o1 \| \| ERNIE Bot 4.0 \| with the guidelines \| You are now a nephrologist, and you need to reference the given ADA guidelines to write an educational handout for patients. The handout should answer the following questions and be written at a sixth-grade reading level. 1.What is early diabetic kidney disease? 2.What are the early signs of diabetic kidney disease? 3.How is diabetic kidney disease treated? 4.What are the risk factors for diabetic kidney disease? 5.How can diabetic kidney disease be prevented? \| \| GPT-4o \| \| ChatGLM4 \| |
| --- | --- | --- | --- | --- | --- | --- | --- | --- | --- | --- | --- | --- |

| **Supplementary Table 2. Patient Assessment Questionnaire** | | | | | |
| --- | --- | --- | --- | --- | --- |
| **Patient comprehensibility scoring criteria** | **Score** |  |  |  |  |
| Is the language of the answer simple and easy to understand? | 1: Very difficult to understand 2: A little hard to understand 3: General 4: Easier to understand 5: Very easy to understand |  |  |  |  |
| Is there jargon in the answer? | 1: A lot 2: There are some 3: General 4: Very little 5：not have |  |  |  |  |
| Is the structure of the answer clear? | 1: Very confusing 2: Some confusion 3: General 4: Clearer 5: Very clear |  |  |  |  |
| Are you prone to following the logic of the answers? | 1: Very difficult 2: A little difficult 3: General 4: Easier 5: Very easy |  |  |  |  |
| Do the answers help you understand the medical knowledge involved? | 1: Not at all 2: A little help 3: General 4: More helpful 5: Very helpful |  |  |  |  |
| **Completeness scoring criteria** | **Score** |  |  |  |  |
| Does the answer contain the main information you want to know? | 1: A lot of information is missing 2: There is some important information missing 3: Complete information |  |  |  |  |
| Does the answer provide an adequate explanation of the relevant issues? | 1: Little explanation 2: Explanation of general 3: Adequate explanations |  |  |  |  |
| Do you think the answer provides sufficient examples or illustrations? | 1: No examples 2: Some examples 3: Sufficient examples |  |  |  |  |
| **Safety Scoring Criteria** | **Score** |  |  |  |  |
| Does the answer mention potential risks or side effects? | 1: No mention 2: A number of references were made 3: All relevant risks are mentioned |  |  |  |  |
| Does the answer suggest that you consult a doctor or professional? | 1: No recommendatio-n 2: Some recommendations 3: Clear recommendatio-ns |  |  |  |  |
| Do the answers contain any information that could be misleading? | 1: A lot of misleading information 2: There is some misdirection 3: No misleading information |  |  |  |  |


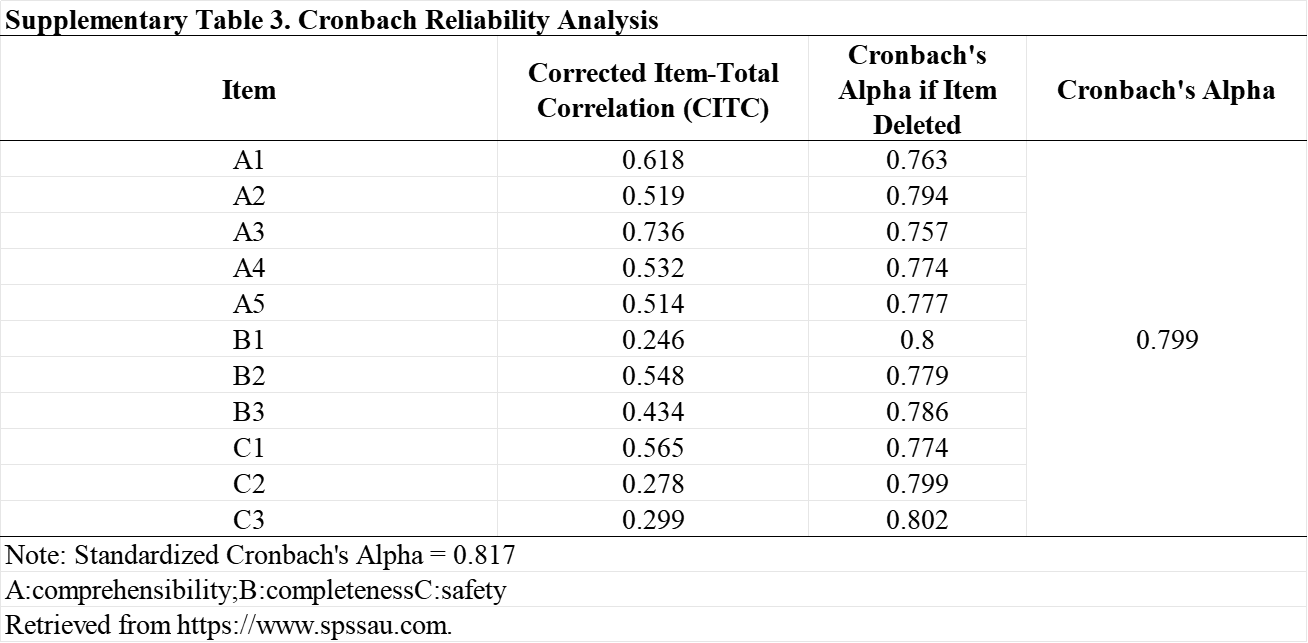


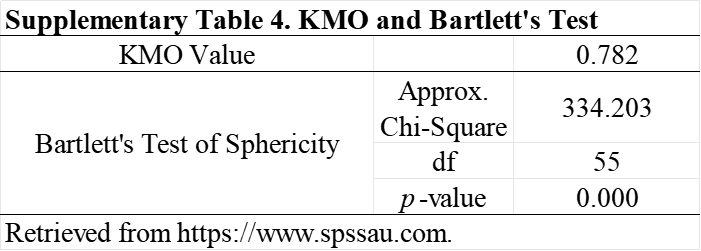


# Supplementary Data

**Patient education materials(PEMs)**

**Not based on guideline version of PEMs**

**ERNIE Bot 4.0:**

**Patient Education Handout: Diabetic Kidney Disease**

**1. What is Early Diabetic Kidney Disease?**

Early diabetic kidney disease, also known as diabetic nephropathy, is when your kidneys start to have problems because of your diabetes. At this stage, your kidneys may let small amounts of protein leak into your urine, but they can still do their job of filtering waste and extra water from your blood. This early stage is important to catch because there are ways to help prevent the disease from getting worse.

**2. What are the Early Signs of Diabetic Kidney Disease?**

In early diabetic kidney disease, you may not feel any different. But, some people may notice:

- They need to urinate more often, especially at night.
- Their urine may have more bubbles than usual.
- They feel tired or their skin is starting to swell, especially around the eyes, ankles, or feet.
- They have high blood pressure.
- It's important to talk to your doctor if you notice any of these signs.

**3. How is Diabetic Kidney Disease Treated?**

Treating diabetic kidney disease focuses on controlling your diabetes and high blood pressure. Your doctor may recommend:

- Taking medicines to lower your blood sugar and keep it in a healthy range. This can include insulin shots or pills.
- Eating a healthy diet low in salt, fat, and sugar.
- Being more active to help your body use insulin better and lower your blood pressure.
- Taking medicines to lower your blood pressure if it's too high.

**4. What are the Risk Factors for Diabetic Kidney Disease?**

Having diabetes is the main risk factor for diabetic kidney disease. But, some things can make it more likely to happen, like:

- High blood sugar levels over a long time.
- High blood pressure.
- Smoking.
- Being overweight.
- Having a family history of kidney disease.

**5. How Can Diabetic Kidney Disease Be Prevented?**

The best way to prevent diabetic kidney disease is to manage your diabetes well. Here's how:

- Check your blood sugar regularly and keep it in your target range.
- Eat healthy foods and stay at a healthy weight.
- Be active and exercise regularly.
- Take all your diabetes medicines as prescribed.
- Get regular check-ups with your doctor, including kidney function tests.
- Avoid smoking.

Remember, diabetic kidney disease can be managed or even prevented if caught early. It's essential to partner with your healthcare team and follow their recommendations. Stay healthy and take care of your kidneys!

中文版：

**患者教育手册：糖尿病肾病**

**1. 什么是早期糖尿病肾病？**

早期糖尿病肾病，也称为糖尿病性肾病，是指由于糖尿病而导致肾脏出现问题的状况。在这个阶段，肾脏可能会让少量的蛋白质漏入尿液中，但它们仍然可以执行过滤血液废物和多余水分的工作。这个早期阶段很重要，因为有一些方法可以帮助防止疾病恶化。

**2. 早期糖尿病肾病的征兆是什么？**

在早期糖尿病肾病中，你可能不会感觉有任何不同。但是，有些人可能会注意到：

- 他们需要更频繁地排尿，尤其是晚上。
- 他们的尿液可能比平时有更多的泡沫。
- 他们感到疲倦，或者他们的皮肤开始肿胀，尤其是眼睛、脚踝或脚部周围。
- 他们有高血压。

如果你注意到这些征兆，务必与医生沟通。

**3. 如何治疗糖尿病肾病？**

糖尿病肾病的治疗主要集中在控制糖尿病和高血压。医生可能会建议：

- 服用药物来降低血糖并保持在健康范围内。这可能包括注射胰岛素或服用药丸。
- 采用低盐、低脂肪和低糖的健康饮食。
- 增加身体活动，帮助身体更好地利用胰岛素并降低血压。
- 如果血压过高，则服用药物来降低。

**4. 糖尿病肾病的风险因素是什么？**

患有糖尿病是糖尿病肾病的主要风险因素。但是，还有一些因素可能使其更容易发生，例如：

- 长期的高血糖水平。
- 高血压。
- 吸烟。
- 超重。
- 有肾脏疾病的家族史。

**5. 如何预防糖尿病肾病？**

预防糖尿病肾病的最好方法是妥善管理糖尿病。以下是一些方法：

- 定期检查血糖并保持在目标范围内。
- 健康饮食并保持健康体重。
- 经常活动并定期锻炼。
- 按医嘱服用所有糖尿病药物。
- 定期接受医生检查，包括肾功能测试。
- 避免吸烟。

请记住，如果及早发现，糖尿病肾病是可以控制甚至预防的。与医疗团队合作并遵循他们的建议至关重要。保持健康，照顾好你的肾脏！

**GPT-4o：**

**Understanding Early Diabetic Kidney Disease: A Simple Guide**

**1. What is Early Diabetic Kidney Disease?**

Early diabetic kidney disease happens when diabetes starts to damage your kidneys. Your kidneys filter waste and extra water from your blood, but high blood sugar can hurt these filters. Over time, this damage can lead to more serious kidney problems.

**2. What Are the Early Signs of Diabetic Kidney Disease?**

In the early stages, you might not feel any different. However, some warning signs include:

- Swelling in your hands, feet, or face (this is called edema)
- High blood pressure
- Feeling tired more often than usual
- Changes in how much you pee (more or less than usual)

Your doctor can also find early signs through blood and urine tests, even if you don't notice any symptoms.

**3. How Is Diabetic Kidney Disease Treated?**

Treatment focuses on controlling your blood sugar and blood pressure. This helps slow down the damage to your kidneys. Some treatments include:

- Medications to lower blood sugar (like insulin or pills)
- Medications to control blood pressure (ACE inhibitors or ARBs)
- Eating a healthy diet that's low in salt and protein
- Regular check-ups to monitor your kidney function

**4. What Are the Risk Factors for Diabetic Kidney Disease?**

Certain factors make it more likely to develop diabetic kidney disease, such as:

- Having diabetes for many years
- Poor blood sugar control
- High blood pressure
- Being overweight
- Smoking
- Family history of kidney disease

**5. How Can Diabetic Kidney Disease Be Prevented?**

The best way to prevent kidney damage is to manage your diabetes carefully. Here are some tips:

- Keep your blood sugar levels in the target range set by your doctor.
- Monitor your blood pressure regularly and take your medication as prescribed.
- Eat healthy, exercise, and maintain a healthy weight.
- Avoid smoking, as it can make kidney damage worse.
- Have regular check-ups with your doctor to catch any early signs of kidney problems.

Taking these steps can help protect your kidneys and keep you healthier for longer!

**中文版：**

**了解早期糖尿病肾病：简单指南**

**1. 什么是早期糖尿病肾病？**

早期糖尿病肾病是指糖尿病开始损害肾脏的情况。肾脏的功能是过滤血液中的废物和多余的水分，但高血糖会损伤这些过滤器。随着时间的推移，这种损害可能导致更严重的肾脏问题。

**2. 早期糖尿病肾病有哪些早期症状？**

在早期阶段，您可能感觉不到任何变化。然而，有一些警告信号包括：

- 手、脚或脸部的肿胀（称为水肿）
- 高血压
- 经常感到疲倦
- 排尿量的变化（比平时更多或更少）

即使您没有注意到症状，医生也可以通过血液和尿液检查发现早期迹象。

**3. 糖尿病肾病是如何治疗的？**

治疗的重点是控制血糖和血压，以减缓肾脏的损害。一些常见的治疗方法包括：

- 降低血糖的药物（如胰岛素或口服药）
- 控制血压的药物（ACE抑制剂或ARB类药物）
- 低盐和低蛋白饮食
- 定期检查以监测肾功能

**4. 糖尿病肾病的危险因素有哪些？**

某些因素会增加患糖尿病肾病的风险，包括：

- 糖尿病病史较长
- 血糖控制不佳
- 高血压
- 超重
- 吸烟
- 家族有肾病史

**5. 如何预防糖尿病肾病？**

预防肾损伤的最佳方法是仔细管理您的糖尿病。以下是一些建议：

- 保持血糖水平在医生设定的目标范围内。
- 定期监测血压并按医嘱服药。
- 健康饮食、锻炼身体并保持健康体重。
- 避免吸烟，因为它会加重肾损伤。
- 定期看医生，及时发现任何肾脏问题的早期迹象。

采取这些措施可以帮助保护您的肾脏，让您保持更长时间的健康！

**ChatGLM4:**

**Understanding Early Diabetic Nephropathy**

**1. What is early diabetic nephropathy?**

Early diabetic nephropathy is a condition that occurs when high blood sugar caused by diabetes injures the tiny blood vessels in the kidneys. The kidneys act as filters in our bodies, removing waste products. Over time, diabetes may make these filters work less efficiently, which can lead to more serious kidney problems.

**2. What are the early signs of diabetic kidney disease?**

In the early stages, you may not feel any signs because the kidneys can still do their job. However, here are some of the things your doctor will look out for Small amounts of protein in the urine (pee), which can be detected by a urine test. High blood pressure, as the kidneys help control blood pressure. Elevated levels of waste products in the blood, which indicates poor kidney filtration.

1. **How is diabetic kidney disease treated?**

Treatment focuses on controlling blood sugar and blood pressure to keep the kidneys healthy. Here's what your doctor may do:

-  Medication: To help control blood sugar and blood pressure.
-  Diet changes: You may need to reduce your intake of salt, protein, and fluids.
-  Lifestyle changes: Regular exercise and not smoking can help.
-  Blood Sugar Checks: Keeping your blood sugar levels in a healthy range is key.

**4. What are the risk factors for diabetic kidney disease?**

**If you have diabetes, the following may increase your risk of developing kidney disease:**

-  Uncontrolled blood sugar or blood pressure.
-  Smoking.
-  Being overweight.
-  A family history of kidney disease.
-  Being older than 60.
-  African American, Hispanic, Native American, or Asian American.

**5. How can I prevent diabetic kidney disease?**

Preventing diabetic kidney disease means taking good care of your diabetes. Here's how to do it:

-  Check your blood sugar regularly: Keep your blood sugar in the range recommended by your doctor.
-  Control your blood pressure: Aim to keep your blood pressure below 140/90 mmHg.
-  Eat a healthy diet: Choose foods low in salt and fat and high in fiber.
-  Stay active: Exercise at least 30 minutes a week, most days.
-  Don't smoke: Smoking is bad for your kidneys and diabetes.
-  See your doctor regularly: Get checked for kidney problems at least once a year.

Following these steps can help protect your kidneys and your health. If you have any questions or concerns about your kidneys or diabetes, always talk to your doctor.

中文版：

**了解早期糖尿病肾病**

**1. 什么是早期糖尿病肾病？**

早期糖尿病肾病是当糖尿病引起的高血糖伤害到肾脏中的微小血管时发生的状况。肾脏就像我们体内的过滤器，清除废物。随着时间的推移，糖尿病可能会使这些过滤器的工作效率降低，这可能导致更严重的肾脏问题。

**2. 早期糖尿病肾病的迹象有哪些？**

在早期，您可能感觉不到任何迹象，因为肾脏仍然可以完成它们的工作。但是，以下是一些医生会注意的事项：

- 尿液（小便）中出现少量蛋白质，这可以通过尿液测试发现。
- 高血压，因为肾脏有助于控制血压。
- 血液中废物产品水平升高，这表明肾脏过滤功能不佳。

**3. 糖尿病肾病如何治疗？**

治疗的重点是控制血糖和血压，以保持肾脏健康。以下是医生可能会做的事情：

- **药物治疗：** 帮助控制血糖和血压。
- **饮食改变：** 您可能需要减少盐分、蛋白质和液体的摄入。
- **生活方式改变：** 定期锻炼和不吸烟可以帮助。
- **血糖检查：** 保持血糖水平在健康范围内是关键。

**4. 糖尿病肾病的风险因素有哪些？**

如果您患有糖尿病，以下情况可能会增加患肾病风险：

- 未控制血糖或血压。
- 吸烟。
- 超重。
- 有肾脏疾病的家族史。
- 年龄超过60岁。
- 非洲裔美国人、西班牙裔、美洲原住民或亚洲美国人。

**5. 如何预防糖尿病肾病？**

预防糖尿病肾病就是好好照顾您的糖尿病。以下是如何做到：

- **定期检查血糖：** 保持血糖在医生建议的范围内。
- **控制血压：** 目标是将血压维持在低于140/90毫米汞柱。
- **健康饮食：** 选择低盐低脂、高纤维的食物。
- **保持活跃：** 每周至少锻炼30分钟，大多数日子。
- **不要吸烟：** 吸烟对肾脏和糖尿病都不好。
- **定期看医生：** 至少每年检查一次是否有肾脏问题。

遵循这些步骤，可以帮助保护您的肾脏和健康。如果您对肾脏或糖尿病有任何疑问或担忧，请务必咨询医生。

**ChatGPT-o1：**

**Understanding Early Diabetic Kidney Disease**

**1. What Is Early Diabetic Kidney Disease?**

Early diabetic kidney disease happens when high blood sugar from diabetes starts to harm your kidneys. Kidneys are like tiny filters that clean your blood. When they get damaged, they can't clean your blood as well as they should.

**2. What Are the Early Signs of Diabetic Kidney Disease?**

At first, you might not notice any signs. But as time goes on, you may have:

- Swelling in your hands, feet, or face
- Feeling tired or weak
- Needing to pee more or less than usual
- Trouble sleeping or thinking clearly
- Upset stomach or loss of appetite

**3. How Is Diabetic Kidney Disease Treated?**

Treatments can help slow down kidney damage. These include:

- **Controlling Blood Sugar:** Keeping your blood sugar levels in a healthy range.
- **Managing Blood Pressure:** High blood pressure can harm your kidneys more.
- **Medications:** Doctors may give you medicines to protect your kidneys.
- **Healthy Eating:** Eating less salt and the right amount of protein.
- **Regular Check-Ups:** Visiting your doctor to monitor your kidneys.

**4. What Are the Risk Factors for Diabetic Kidney Disease?**

You might have a higher chance of getting kidney disease if you:

- Have high blood sugar levels
- Have high blood pressure
- Have had diabetes for a long time
- Smoke cigarettes
- Are overweight
- Have family members with kidney disease

**5. How Can Diabetic Kidney Disease Be Prevented?**

You can help protect your kidneys by:

- **Keeping Blood Sugar Controlled:** Check your blood sugar often.
- **Managing Blood Pressure:** Keep your blood pressure in a healthy range.
- **Eating Healthy Foods:** Choose fruits, vegetables, and whole grains.
- **Being Active:** Exercise regularly to stay healthy.
- **Not Smoking:** Smoking can harm your kidneys.
- **Getting Regular Tests:** Have your kidneys checked every year.

**Remember:** Taking care of your diabetes helps take care of your kidneys!

中文版：

**了解早期糖尿病肾病**

**1. 什么是早期糖尿病肾病？**

早期糖尿病肾病是指高血糖开始损害您的肾脏。肾脏就像小滤器，清洁您的血液。当它们受损时，无法像应该的那样有效地清洁血液。

**2. 糖尿病肾病的早期症状是什么？**

一开始，您可能感觉不到任何症状。但随着时间推移，您可能会有：

- 手、脚或脸部肿胀
- 感到疲倦或虚弱
- 排尿次数比平常多或少
- 睡眠困难或思维不清
- 胃部不适或食欲不振

**3. 糖尿病肾病如何治疗？**

治疗可以帮助减缓肾脏损害。包括：

- **控制血糖：** 保持血糖水平在健康范围内。
- **管理血压：** 高血压会进一步损害肾脏。
- **药物治疗：** 医生可能会给您开药来保护肾脏。
- **健康饮食：** 减少盐的摄入，适量食用蛋白质。
- **定期检查：** 定期看医生，监测肾脏健康。

**4. 哪些是糖尿病肾病的风险因素？**

如果您有以下情况，患肾病的风险更高：

- 血糖水平高
- 血压高
- 长期患有糖尿病
- 吸烟
- 超重
- 家族有肾病史

**5. 如何预防糖尿病肾病？**

您可以通过以下方法保护肾脏：

- **控制血糖：** 经常检查血糖。
- **管理血压：** 保持血压在健康范围。
- **健康饮食：** 多吃水果、蔬菜和全谷物。
- **积极运动：** 定期锻炼，保持健康。
- **不吸烟：** 吸烟会损害肾脏。
- **定期检测：** 每年进行肾脏检查。

**记住：照顾好您的糖尿病，就是在保护您的肾脏！**

**Based on guideline version of PEMs**

**ERNIE Bot 4.0:**

**Educational Handout for Patients: Understanding Diabetic Kidney Disease**

**1. What is early diabetic kidney disease?**

Early diabetic kidney disease, also called chronic kidney disease (CKD), is when your kidneys start to lose some of their ability to work properly. This usually happens slowly over many years because of high blood sugar levels from diabetes. If not treated well, it can get worse and lead to more serious problems.

**2. What are the early signs of diabetic kidney disease?**

Sometimes, early diabetic kidney disease doesn't have many noticeable signs. But here are some things to look out for:

- More frequent urination, especially at night
- Swelling in your legs, ankles, or feet
- Feeling very tired or weak
- Trouble concentrating
- Loss of appetite

To catch early diabetic kidney disease, it's important to get regular check-ups, even if you don't have any symptoms.

**3. How is diabetic kidney disease treated?**

Treating diabetic kidney disease involves managing your diabetes and blood pressure. Here are some key treatments:

- **Controlling Blood Sugar**: Keeping your blood sugar levels within a healthy range can help slow down kidney damage.
- **Controlling Blood Pressure**: High blood pressure can harm your kidneys. Medicines like ACE inhibitors or ARBs can help lower your blood pressure and protect your kidneys.
- **Lifestyle Changes**: Eating a healthy diet, staying active, and quitting smoking can also help manage your diabetes and protect your kidneys.
- **Medicines**: Sometimes, special medicines like SGLT2 inhibitors or GLP-1 receptor agonists can help reduce the risk of kidney disease getting worse.

**4. What are the risk factors for diabetic kidney disease?**

Having certain things can increase your chances of getting diabetic kidney disease:

- Having diabetes for a long time
- Not controlling your blood sugar well
- Having high blood pressure
- Being overweight or obese
- Having a family history of kidney disease
- Being African American, Native American, Asian American, or Pacific Islander

**5. How can diabetic kidney disease be prevented?**

Preventing diabetic kidney disease involves taking good care of your diabetes and your overall health. Here are some steps you can take:

- **Manage Your Blood Sugar**: Keep your blood sugar levels under control by taking your medicines as prescribed, eating healthy foods, and staying active.
- **Control Your Blood Pressure**: Maintain a healthy blood pressure by taking any prescribed medicines and making healthy lifestyle choices.
- **Stay Active**: Regular physical activity can help keep your blood sugar and blood pressure in check.
- **Eat Well**: Choose foods that are low in salt, fat, and sugar, and high in fruits, vegetables, and whole grains.
- **Quit Smoking**: Smoking can harm your kidneys and make your diabetes harder to control.
- **Get Regular Check-ups**: See your healthcare provider regularly for blood sugar and blood pressure checks, and kidney function tests.

Remember, the best way to prevent or manage diabetic kidney disease is to work closely with your healthcare team and make healthy lifestyle choices.

中文版：

## 1.什么是早期糖尿病肾病？

早期糖尿病肾病，也称为慢性肾脏病（CKD），是指肾脏开始丧失部分正常功能。这通常是由于多年糖尿病导致的高血糖水平而缓慢发生的。如果治疗不当，病情会恶化并导致更严重的问题。

## 2.糖尿病肾病的早期症状是什么？

有时，早期糖尿病肾病没有太多明显症状。但以下是一些需要注意的事项：

- 排尿更频繁，尤其是夜间
- 腿部、脚踝或脚部肿胀
- 感觉非常疲倦或虚弱
- 注意力不集中
- 食欲不振

要及早发现糖尿病肾病，即使没有症状，定期进行体检也很重要。

## 3.如何治疗糖尿病肾病？

糖尿病肾病的治疗包括控制糖尿病和血压。以下是一些关键治疗方法：

- 控制血糖：将血糖水平保持在健康范围内有助于减缓肾脏损伤。
- 控制血压：高血压会损害肾脏。ACE抑制剂或ARBs等药物有助于降低血压并保护肾脏。
- 改变生活方式：健康饮食、保持活跃和戒烟也有助于控制糖尿病和保护肾脏。
- 药物：有时，特殊药物如SGLT2抑制剂或GLP-1受体激动剂有助于降低肾病恶化的风险。

## 4.糖尿病肾病的风险因素是什么？

具备某些条件会增加患糖尿病肾病的风险：

- 长期患有糖尿病
- 血糖控制不佳
- 患有高血压
- 超重或肥胖
- 有肾病家族史
- 属于非裔美国人、美洲原住民、亚裔美国人或太平洋岛民

## 5.如何预防糖尿病肾病？

预防糖尿病肾病需要妥善控制糖尿病和整体健康状况。以下是一些可以采取的措施：

- 控制血糖：通过按医嘱服药、健康饮食和保持活跃，将血糖水平控制在正常范围内。
- 控制血压：通过服用任何处方药和做出健康的生活方式选择，保持健康的血压。
- 保持活跃：定期进行体育活动有助于控制血糖和血压。
- 健康饮食：选择低盐、低脂肪、低糖以及富含水果、蔬菜和全谷物的食物。
- 戒烟：吸烟会损害肾脏，并使糖尿病更难控制。
- 定期体检：定期找医疗保健提供者进行血糖和血压检查以及肾功能测试。

请记住，预防或管理糖尿病肾病的最佳方法是与医疗保健团队紧密合作并做出健康的生活方式选择。

**GPT-4o：**

**What is Early Diabetic Kidney Disease?**

Diabetic kidney disease happens when high blood sugar from diabetes damages the kidneys. It can lead to kidney failure if not treated early. When kidney disease starts, the kidneys stop filtering waste and extra fluid from your body as well as they should. This can happen slowly over many years.

**What are the Early Signs of Diabetic Kidney Disease?**

The first sign of diabetic kidney disease is usually an increase in a protein called albumin in your urine. This is called albuminuria. You won’t feel sick at this stage, but testing your urine can catch it early. Another sign is a decrease in how well your kidneys filter blood, which can be measured by your doctor with a test called eGFR (estimated glomerular filtration rate).

**How is Diabetic Kidney Disease Treated?**

Treatment focuses on keeping your blood sugar and blood pressure under control. This can help slow the damage to your kidneys. Medications called ACE inhibitors or ARBs are often given to help protect your kidneys, especially if protein is found in your urine. Other treatments include SGLT2 inhibitors, which are newer medications that can help prevent kidney disease from getting worse.

**What are the Risk Factors for Diabetic Kidney Disease?**

You are more likely to get diabetic kidney disease if you:

- Have had diabetes for a long time
- Have high blood pressure
- Are not keeping your blood sugar under control
- Smoke
- Have a family history of kidney disease

**How Can Diabetic Kidney Disease Be Prevented?**

You can help prevent kidney disease by managing your diabetes well. Here are some important steps:

- Keep your blood sugar in your target range
- Control your blood pressure
- Eat a healthy diet that is low in salt and protein
- Exercise regularly
- Don’t smoke
- Get regular check-ups with your doctor, including urine tests to check for protein and blood tests to check kidney function

中文版：

**什么是早期糖尿病肾病？**

糖尿病肾病是指由于糖尿病导致的高血糖损害了肾脏。如果不及时治疗，可能会导致肾衰竭。当肾病开始时，肾脏无法像以前一样有效地过滤体内的废物和多余的液体。这种情况通常会在多年内逐渐发生。

**糖尿病肾病的早期症状是什么？**

糖尿病肾病的第一个症状通常是尿液中蛋白质（称为白蛋白）的增加，这被称为白蛋白尿。你可能在这个阶段不会感到不适，但通过尿检可以早期发现。另一个症状是肾脏过滤血液的能力下降，这可以通过医生的检测来评估，称为eGFR（估算肾小球滤过率）。

**糖尿病肾病如何治疗？**

治疗重点是控制血糖和血压，这有助于减缓肾脏损伤。通常会使用ACE抑制剂或ARB类药物，尤其是当尿液中发现蛋白质时，这些药物有助于保护肾脏。其他治疗包括SGLT2抑制剂，这是一种新型药物，可以帮助防止肾病恶化。

**糖尿病肾病的风险因素有哪些？**

以下情况会增加你患糖尿病肾病的风险：

- 患糖尿病时间较长
- 有高血压
- 血糖控制不佳
- 吸烟
- 有家族肾病史

**如何预防糖尿病肾病？**

通过良好管理糖尿病，可以帮助预防肾病。以下是一些重要步骤：

- 将血糖保持在目标范围内
- 控制血压
- 保持低盐低蛋白的健康饮食
- 经常锻炼
- 不吸烟
- 定期看医生，包括尿检以检测蛋白质和血液检测以检查肾功能

**ChatGLM4:**

**1. What is early diabetic kidney disease**?

Early diabetic kidney disease is when your kidneys start to have problems because of diabetes. Your kidneys filter waste from your blood, but when you have diabetes, they can’t do this as well.

**2. What are the early signs of diabetic kidney disease**?

There are no obvious signs of early diabetic kidney disease. It often starts without you noticing anything is wrong. However, some people might notice they have more urine or they need to go to the bathroom more often.

**3. How is diabetic kidney disease treated**?

To treat early diabetic kidney disease, doctors focus on managing your diabetes and blood pressure. This can help slow down the damage to your kidneys. Some medicines can also help protect your kidneys.

**4. What are the risk factors for diabetic kidney disease**?

There are some things that can increase your chances of getting diabetic kidney disease:

- Having type 1 or type 2 diabetes
- Having high blood pressure
- Having a family history of kidney problems
- Being overweight
- Not getting enough exercise

**5. How can diabetic kidney disease be prevented**?

The best way to prevent diabetic kidney disease is to:

- Keep your diabetes under control by managing your blood sugar and A1C levels
- Keep your blood pressure low
- Eat a healthy diet
- Exercise regularly
- Maintain a healthy weight

**Remember, it’s important to talk to your doctor if you have diabetes or are at risk for diabetic kidney disease. They can help you monitor your kidneys and manage your diabetes and blood pressure to keep your kidneys healthy**.

中文版：

**1. 什么是早期糖尿病肾病**？

早期糖尿病肾病是指由于糖尿病而导致的肾脏问题开始出现。你的肾脏负责过滤血液中的废物，但当你患有糖尿病时，它们无法正常工作。

**2. 早期糖尿病肾病的早期迹象有哪些**？

早期糖尿病肾病通常没有明显的迹象。它往往开始时你并没有察觉到任何问题。然而，有些人可能会发现他们尿量增多，或者需要更频繁地上厕所。

**3. 如何治疗糖尿病肾病**？

治疗早期糖尿病肾病的关键是管理好你的糖尿病和血压。这可以帮助减缓肾脏受损的速度。有些药物也可以帮助保护你的肾脏。

**4. 糖尿病肾病的危险因素有哪些**？

以下是一些可能会增加你患糖尿病肾病的风险的因素：

- 患有1型或2型糖尿病
- 血压高
- 家族有肾脏问题史
- 超重
- 缺乏运动

**5. 如何预防糖尿病肾病**？

预防糖尿病肾病最好的方法是：

- 通过管理好你的血糖和A1C水平来控制好你的糖尿病
- 保持血压低
- 吃健康的饮食
- 常锻炼
- 保持健康的体重

**请记住，如果你患有糖尿病或患有糖尿病肾病的风险，请务必与医生交谈。他们可以帮助你监测你的肾脏，并管理好你的糖尿病和血压，以保持你的肾脏健康**。

**Physician-generated PEM:**

**1.What is Early Diabetic Nephropathy?**

Early diabetic nephropathy is the early stage of diabetic nephropathy, there will be persistent microalbuminuria, after treatment some patients can be reversed.

**2. What are the early signs of diabetic nephropathy?**

Early manifestation of diabetic nephropathy may not be obvious, the glomerulus is the “filter” in the kidney, at this time the glomerular filtration rate is mildly increased, with the prolongation of the course of the disease, there will be persistent microalbuminuria, increase in urinary foam, and the glomerular filtration rate is normal or mildly decreased.

**3. How to treat diabetic nephropathy?**

(1) Lifestyle changes

a. Healthy diet

High-quality low-protein diet: in the early stage of diabetic nephropathy, you can choose high-quality low-protein food, which helps to reduce the burden on the kidneys; limit sodium intake: if you have edema or high blood pressure, you have to reduce the salt intake and choose low-salt diet, which helps to control the blood pressure.

b. Exercise

According to your physical condition, take reasonable and regular physical exercise, such as walking, swimming or cycling.

c.Smoking cessation

Smoking is a risk factor for the progression of kidney function in DM patients. Reducing smoking or quitting smoking is an important measure for diabetic patients to prevent or control the progression of diabetic nephropathy.

d. Maintain a healthy weight

(2) Control blood sugar

Strict control of blood glucose level and keeping blood glucose within a reasonable range can delay the onset and progression of diabetic nephropathy. The following medications can be used:

Traditional diabetes drugs (e.g. insulin and metformin)

Newer diabetes drugs (e.g. GLP-1 receptor agonists and SGLT2 inhibitors)

(3) Blood pressure control

Blood pressure goal: It is recommended to control blood pressure at <130/80 mmHg.

Recommended medications: Angiotensin-converting enzyme (ACE) inhibitors and angiotensin 2 receptor blockers (ARBs) are recommended as first-line therapeutic agents.

(4) Lipid control

Use of statins: can help lower cholesterol levels while reducing urinary protein and protecting kidney health.

(5) Others

Fenetyllone is an effective drug which can reduce high glomerular pressure and hyperfiltration, thus reducing the excretion of urinary protein, it not only reduces the risk of cardiovascular events, but also slows down the progression of kidney disease. In addition, proprietary Chinese medicine can also be used as an adjunctive treatment for diabetic nephropathy, providing more choices and support for patients.

**4. What are the risk factors for diabetic nephropathy?**

Irreversible risk factors: age, gender, race, family history and duration of diabetes mellitus

Modifiable risk factors: hyperglycemia, hypertension, albuminuria, dyslipidemia and smoking

**5. How to prevent diabetic nephropathy?**

① Good control of blood glucose and blood pressure to keep them in the ideal range.

② Regular monitoring: Check blood glucose, blood pressure and microalbuminuria regularly for timely detection of problems and early intervention.

③ Treatment of diabetes: treat diabetes systematically under the guidance of a doctor's scientific and standardized instructions, and effectively prevent and treat diabetic nephropathy.

④ Healthy lifestyle: Maintain a healthy weight, moderate exercise, quit smoking, and strictly control blood lipids to reduce the risk of diabetic nephropathy.

中文版：

**患者教育材料**

1. **什么是早期糖尿病肾病？**

早期糖尿病肾病是糖尿病肾病的早期阶段，会有持续性微量白蛋白尿出现，进行治疗后部分患者是可以逆转的。

1. **糖尿病肾病的早期表现有哪些？**

糖尿病肾病的早期表现可能不明显，肾小球是肾脏中的“过滤器”，此时肾小球滤过率轻度增高，随着病程的延长，会出现持续微量白蛋白尿，尿中泡沫增多，肾小球滤过率正常或轻度下降。

1. **如何治疗糖尿病肾病？**
2. **改变生活方式**
3. 健康饮食

优质低蛋白饮食：在糖尿病肾病早期，可以选择高质量的低蛋白食物，有助于减轻肾脏负担；限制钠盐摄入：如果有水肿或高血压，要减少盐的摄入，选择低盐饮食，帮助控制血压。

1. 运动

根据自己的身体情况，进行合理、规律的体育锻炼，比如散步、游泳或骑自行车。

1. 戒烟

吸烟是DM患者肾功能进展的危险因素，减少吸烟或戒烟是糖尿病患者预防或控制糖尿病肾病进展的重要措施。

1. 保持健康体重
2. **控制血糖**

严格控制血糖水平，保持血糖在合理范围内，可以延缓糖尿病肾病的发生和进展。可以使用以下药物：

传统糖尿病药物（如胰岛素和二甲双胍）

新型糖尿病药物（如GLP-1受体激动剂和SGLT2抑制剂）

1. **控制血压**

血压目标：建议将血压控制在<130/80 mmHg。

推荐用药：血管紧张素转换酶（ACE）抑制剂和血管紧张素2受体阻滞剂（ARB）被推荐作为一线治疗药物。

1. **控制血脂**

使用他汀类药物：可以帮助降低胆固醇水平，同时减少尿蛋白，保护肾脏健康。

1. **其他**

非奈利酮是一种有效的药物，该药可以降低肾小球的高压和高滤过，从而减少尿蛋白的排出，它不仅可以降低心血管事件的风险，还能延缓肾病的进展。此外，中成药也可以作为糖尿病肾病的辅助治疗方法，为患者提供更多的选择和支持。

1. **糖尿病肾病的风险因素是什么？**

不可逆的风险因素：年龄、性别、种族、家族史和糖尿病的持续时间

可改变的风险因素：高血糖、高血压、白蛋白尿、血脂异常和吸烟

1. **如何预防糖尿病肾病？**

①良好控制血糖和血压，保持血糖和血压在理想范围。

②定期监测：定期检查血糖、血压以及微量白蛋白尿，以便及时发现问题并进行早期干预。

③治疗糖尿病：在医生科学规范指的指导下系统治疗糖尿病，有效防治糖尿病肾病。

④健康生活方式：保持健康体重，适量运动，戒烟，严格控制血脂，以降低糖尿病肾病发生风险。
